# Histochemistry for Molecular Imaging in Nanomedicine

**DOI:** 10.3390/ijms25158041

**Published:** 2024-07-24

**Authors:** Manuela Malatesta

**Affiliations:** Department of Neurosciences, Biomedicine and Movement Sciences, University of Verona, I-37134 Verona, Italy; manuela.malatesta@univr.it

**Keywords:** antibodies, cultured cells, histochemical stains, immunofluorescence, immunohistochemistry, in vivo models, nanoparticles, tissue slice

## Abstract

All the nanotechnological devices designed for medical purposes have to deal with the common requirement of facing the complexity of a living organism. Therefore, the development of these nanoconstructs must involve the study of their structural and functional interactions and the effects on cells, tissues, and organs, to ensure both effectiveness and safety. To this aim, imaging techniques proved to be extremely valuable not only to visualize the nanoparticles in the biological environment but also to detect the morphological and molecular modifications they have induced. In particular, histochemistry is a long-established science able to provide molecular information on cell and tissue components in situ, bringing together the potential of biomolecular analysis and imaging. The present review article aims at offering an overview of the various histochemical techniques used to explore the impact of novel nanoproducts as therapeutic, reconstructive and diagnostic tools on biological systems. It is evident that histochemistry has been playing a leading role in nanomedical research, being largely applied to single cells, tissue slices and even living animals.

## 1. Introduction

Nanomedicine is the application of nanotechnology to medicine. It is a young science, the term “nanomedicine” making its first official appearance in 1999 [1,2]. Despite its short history, nanomedical research has given origin to various application fields, such as nanodiagnostics (e.g., nanoimaging, nanobiosensors), nanopharmaceuticals (e.g., nanovectors for drugs or nucleic acids delivery, nanovaccines), regenerative nanomedicine (e.g., nanoimplants, exosomes), nanosurgery (e.g., nanoparticles (NPs) activated by external energy source), and nanorobotics (e.g., nanorobots for cancer treatment or tissue repair). All these nanotechnological devices have to deal with the common requirement of facing the complexity of a living organism. Therefore, the development of nanoconstructs designed for medical purposes must necessarily involve the study of their structural and functional interactions and the effects on cells, tissues, and organs, to ensure both effectiveness and safety. To this aim, imaging techniques proved to be extremely valuable to visualize the NPs in the biological environment and detect the morphological and molecular modifications they have induced. In particular, imaging techniques proved to be especially useful to understand the mechanism(s) allowing NPs to enter the cells: most NPs cross the plasma membrane by endocytosis, entering the cell enclosed in endosomes, but lipid-based or lipid-coated NPs may cross the membranes without endosomes, likely fusing with the plasma membrane. Imaging techniques were also crucial to clarify the intracellular fate of the internalized NPs, revealing if NP-containing endosomes fuse with lysosomes leading to the nanoconstruct degradation or NPs’ escape endosomes, thus occurring free in the cytosol and slowing down their degradation even for long times (reviewed in [3]).

The novel needs of nanomedical research have fostered the advancement of modern imaging technology [4,5,6,7,8] but have also led to a sort of renaissance of classical imaging techniques [3,9,10]. In particular, histochemistry is a long-established science able to provide molecular information on cell and tissue components in situ, bringing together the potential of biomolecular analysis and imaging. These features have made histochemistry a widely used approach in nanomedical research [11].

The present review article aims at offering an overview of the various histochemical techniques used to explore the impact of novel nanoconstructs on biological systems, highlighting their central role in the advancement of nanomedicine. Attention has been paid to studies dealing with the development of nanoproducts as therapeutic, reconstructive, and diagnostic tools, whereas merely toxicological studies were excluded.

## 2. Histochemistry: Tradition for Novelty

In the Merriam-Webster dictionary, histochemistry is the term defining “a science that combines the techniques of biochemistry and histology in the study of the chemical constitution of cells and tissues” (https://www.merriam-webster.com/dictionary/histochemistry#h1 (accessed on 8 June 2024)); similarly, in the English Oxford Dictionary (https://en.oxforddictionaries.com/definition/histochemistry (accessed on 8 June 2024)), histochemistry is “the study of the chemical constituents and properties of tissues and cells, typically by the use of special staining methods; a branch of histology dealing with this”. These definitions seem to indicate that the main function of histochemistry is to statically describe the chemistry of tissues’ and cells’ structures. Actually, this was the scientists’ attitude at the end of the 19th century and for some decades on, when new dyes and staining methods were used to better describe morphology of tissues and cells by light microscopy, and not to identify specific chemical components [12]. Only in the first half of the 20th century, Lucien Lison [13] and David Glick [14], in their classical textbooks, presented histochemistry as the discipline able to elucidate the biochemical features of tissues and cells by specific chemical reactions on histological sections. At that time, two milestones were established to identify molecules in situ: the DNA-specific Feulgen reaction and the application of fluorochrome-conjugated antibodies as histochemical tools. The first one [15], which—as Frederick H. Kasten [16] wrote—“may be regarded as the first truly histochemical reaction”, paved the way for a substantial amount of literature on quantitative assessment by microphotometry of the DNA content in normal and diseased tissues. On the other hand, immunohistochemistry was proposed as a technique using antibodies to detect antigens in situ on tissue sections. Fluorescein isothiocyanate (FITC)-labelled antibodies were originally used to reveal bacterial antigens in infected tissues [17]. Thanks to the improvement of protein conjugation, enzymes such as peroxidase and alkaline phosphatase were then also used as antibody markers [18], while the labelling of antibodies with electron-dense substances (e.g., colloidal gold) opened the way to ultrastructural immunohistochemistry that allows identifying antigens at the sub-cellular level, revealing features which are indiscernible by light microscopy [19]. The possibility to detect the final reaction products by different imaging techniques presently makes immunohistochemistry the most broadly used technique in basic and applied biomedical research.

In 1969, Mary-Lou Pardue and Joseph G. Gall first proposed the technique of radioactive in situ hybridization, which became increasingly popular after non-radioactive probes had been introduced (reviewed in [20]). A metabolic mapping of tissues may be obtained by enzyme histochemistry on cryosections: by this method, biochemistry and morphology are effectively linked, as an occurring enzyme activity is topographically located. Enzyme histochemistry thus allows us to notice cell metabolic changes occurring as a consequence of experimental treatments or pathologies, even in the absence of evident changes in the histological or immunohistochemical features [21].

Actually, over the last 70 years, histochemistry has remarkably progressed, and specific and sensitive techniques have been developed for visualizing dynamic events, with special attention to the detection of single molecules in the very place where they exert their functions in live cells [22,23]. In situ hybridization and especially immunohistochemistry have widely been applied to label and locate specific nucleic acid sequences or proteins, while multiple techniques have been set up for the simultaneous detection of different molecular species in situ. In particular, the long-established multicolor immunofluorescence assays [24,25] have recently been improved by mass cytometry where rare-earth-metal isotopes with known atomic mass are used to label antibodies, so that up to more than 30 different proteins may be localized in a single tissue section (reviewed in [26]).

Histochemistry has found wide application in nanomedical research. Different histochemical techniques have been used—sometimes simultaneously—to label the nanoconstructs and to detect structural or functional features of the biosystem, thus providing the unique opportunity to dynamically track the fate of the NPs while visualizing their local effects on the histological constituents. The following chapters report numerous examples of studies where histochemical techniques gave essential information for the advancement of knowledge in nanomedical research. The studies were grouped according to the application of histochemical staining only, immunohistochemical techniques only, or a combination of histochemical stains and immunolabelling.

## 3. Histochemical Staining Techniques in Nanomedical Studies

Classical histochemical staining techniques have been applied in often innovative ways to detect NPs inside cells and tissues.

Prussian blue has been the histochemical method of choice to detect iron-based NPs at bright field microscopy in numerous studies in vitro and ex vivo [27,28,29,30,31,32,33,34,35,36,37,38,39,40,41,42,43,44].

**Figure 1:**
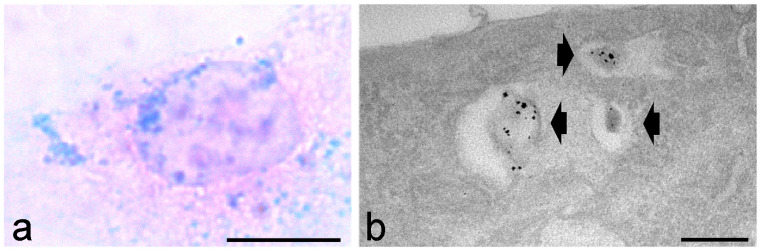
Cultured mouse myoblasts treated with hyaluronic acid-based nanoparticles and stained with the Alcian blue method. (**a**) Bright field microscopy: the nanoparticle aggregates are visible as blue dots; the cell is counterstained with nuclear fast red. (**b**) Transmission electron microscopy: three hyaluronic acid-based nanoparticles enclosed in endosomes (arrowheads) show an electron dense granular dye precipitate. Bars: 10 μm (**a**); 200 nm (**b**). Images from Carton et al. [46].

Alcian blue staining allowed the detection of hyaluronic acid-based NPs [45] in cultured muscle cells at bright field microscopy, thus overcoming the problem of the scarce visibility of fluorescently labelled NPs in highly autofluorescent samples, as well as at transmission electron microscopy (TEM), where the intrinsic low-electron density of these organic NPs makes them hardly recognizable [46,47] (Figure 1).

Silver enhancement was used to make 5 nm gold NPs clearly visible at light microscopy and TEM in rat brain slices [48].

**Figure 2:**
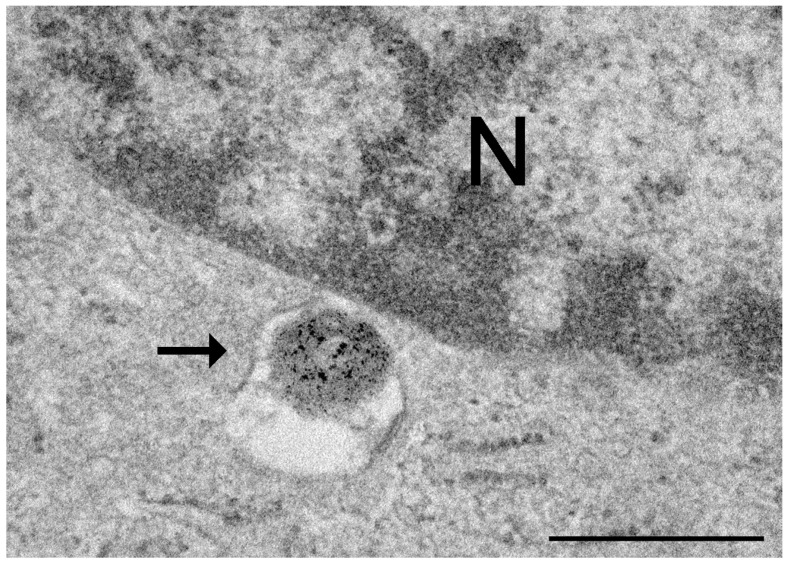
Transmission electron micrograph of a cultured rat neuronal cell treated with fluorescently labelled chitosan-based nanoparticles and submitted to the diaminobenzidine (DAB) photooxidation method. A nanoparticle enclosed in an endosome (arrow) shows the fine granular, electron dense product of DAB oxidation. N, nucleus. Bar: 500 nm. Image from Malatesta et al. [49].

3,3′-Diaminobenzidine (DAB) photooxidation was applied to make fluorophores-labelled NPs visible not only in fluorescence microscopy but also in bright field microscopy and TEM, thanks to the brownish electron-dense DAB precipitates [49,50] (Figure 2).

Histochemical stains were also used to reveal the effect of NP administration. As an example, the targeting to amyloid plaques of gadolinium-based Cy5.5/alkaline phosphatase-labelled NPs functionalized for early diagnosis of amyloidosis was demonstrated with fluorescence microscopy by staining amyloid plaques with Thioflavin-S, while tetramethylrhodamine conjugated phalloidin was used to visualize actin filaments [51].

However, most nanomedical studies made use of immunohistochemical techniques or combined the use of labelled antibodies with classical histochemical stains, as described in points 4 and 5.

## 4. Immunohistochemistry in Nanomedical Studies

Immunohistochemical techniques have been widely applied in nanomedical studies to assess the efficacy of the tested nanoconstructs, using antibodies to detect specific molecules in cells and tissues.

### 4.1. In Vitro Applications

Some studies were carried out on in vitro models, where the immunohistochemical procedures were applied to cultured cells.

Searching for innovative antitumor nanotools, various tight-junction proteins (occluding, zonula occludens-1, claudin-1, α-tubulin) were immunolabelled in cultured colon cancer cells by using antibodies revealed with Alexa^®^ 488-conjugated secondary antibodies, and their redistribution was investigated at fluorescence microscopy following administration of citrate-capped gold NPs loaded with cetuximab [52]. This allowed for confirming the suitability of these NPs to improve paracellular permeability, thus enhancing the therapeutic efficacy of the loaded anticancer agent.

In another study, the suitability of chitosan NPs to deliver the hypometabolizing D-Ala(2)-D-Leu(5)-enkephalin as a potential antitumor agent [53] was assessed in cultured rat neuronal cells by immunolabelling the drug at both fluorescence microscopy and TEM using Alexa Fluor^®^ 594 and colloidal gold particles as probe markers, respectively. Moreover, the effect of the drug on transcription and splicing factors was assessed by immunogold technique. The results proved the presence of drug molecules inside the NPs as well as free in the cell, and a reversible reduction in nuclear activity was observed [54].

The therapeutic potential of nanoconstructs was also explored for infectious diseases. Nanoformulations of various crystalline antiretroviral drugs, set up with the aim to reduce disease morbidity and mortality among human immunodeficiency virus (HIV)-infected individuals, were tested for their efficacy in cultured monocyte-derived macrophages by immunostaining the HIV-1p24 antigen with primary antibodies revealed by horseradish peroxidase (HRP)-labelled secondary antibody and DAB staining [55].

Gold, silver, and platinum NPs were screened for antiparasitic activity against *Toxoplasma gondii* using cultured human fibroblasts. The protozoan invasion and intracellular replication were detected by the immunofluorescence labelling of specific parasite antigens using Alexa^®^ fluorophores [56].

In the frame of reconstructive nanomedical research, the suitability of the fungus *Hericium erinaceus* extract to promote functional recovery of nerve injury was tested on cultured rat pheochromocytoma cells using myco-synthesized gold NPs: the immunofluorescence detection of neurofilaments by FITC-conjugated antibodies allowed for the quantification of neurite-bearing cells, thus demonstrating a stimulatory effect of NPs on neurite outgrowth [57].

To investigate the mechanisms by which TiO_2_ nanotubes mitigate the immune response in biomaterial implantations in comparison to flat Ti surfaces, a murine macrophage line was treated with these nanoconstructs, and immunofluorescence microscopy was used to detect the markers of pro-inflammatory stimuli, nuclear factor kappa B (NF-κB) p65 subunit, and phosphorylated IkB-α protein by antibodies revealed with Alexa Fluor^®^ 488- and Alexa Fluor^®^ 546-conjugated secondary probes. This approach allowed to demonstrate that TiO_2_ nanotubes inhibit both NF-κB activation and p65 nuclear translocation [58].

### 4.2. Ex Vivo Applications

More numerous are the studies where the effects of the nanoconstructs were investigated in vivo, and immunohistochemical procedures were applied on tissue slices obtained from the treated animals or human samples.

In the frame of nanomedical research for innovative antitumor therapeutic and diagnostic strategies, immunofluorescence (using Cy3-conjugated antibodies) and immunohistochemistry (using HRP-conjugated antibodies revealed with DAB) for the endothelial marker CD31 and the intercellular adhesion molecule-1 were performed on mouse gastric tumor sections to test the antitumor activity of poly-lactic-co-glycolic acid NPs coated with T-lymphocyte membranes. The probes demonstrated tumor targeting of NPs as well as an upregulation of the expression of adhesion molecules in tumor vessels, which likely contributed to the accumulation of NPs in these sites [59]. Immunohistochemical detection of CD31 also demonstrated a significant reduction in microvascular density in mice colon tumors after systemic treatment with poly(ethylene glycol) modified poly(DL-lactide-co-glycolide) NPs loaded with the recombinant human endostatin, endostar [60].

To demonstrate the translational efficacy of the C-X-C chemokine receptor type 4 (CXCR4)- small interfering RNAs (siRNAs)/dextran-spermine NPs in a mouse colon carcinoma cell line systemically administered to a mouse model of colorectal cancer, immunohistochemistry for the presence of CXCR4 was performed on distal ileum and colon slices using HRP-conjugated probes revealed with DAB. The reduced expression of CXCR-4 in tumor cells was interpreted as a positive result since this receptor plays a key role in cell metastasis [61].

Sections of mouse tumor tissue were immunohistochemically processed for Ki-67 (a proliferation marker), CD31, and the vascular endothelial growth factor, VEGF (a protein promoting angiogenesis), to explore the antitumor effects on human cervical carcinoma by a synergistic approach consisting of methoxy poly(ethylene glycol)–poly(ε-caprolactone) NPs loaded with paclitaxel (a widely used chemotherapy agent) and radiotherapy. The antibodies, conjugated with biotin and revealed with DAB, demonstrated a decrease in Ki-67-positive cells and immunoreactive microvessels as well as a lower expression of VEGF, thus proving the suitability of this nanotechnological approach [62].

Immunofluorescence and immunohistochemistry were used to investigate the antitumor activity of polymeric NPs loaded with paclitaxel and coated with the monoclonal antibody MECA79, which recognizes the high-endothelial venules that form in human pancreatic ductal adenocarcinoma. Mouse pancreatic tumor tissues were immunolabelled for peripheral node addressin (the protein specifically expressed by high-endothelial venules), CD31, caspase-3 (as a typical hallmarks of apoptosis), collagen I and IV, fibronectin, α-smooth muscle actin, human cutaneous lymphocyte antigen, human leukocyte antigen class I, and Ki-67. Results showed that this treatment improved the delivery of the drug at the tumor site, reducing tumor size and vascularization, and increasing the apoptosis rate [63].

Abraxane NPs (albumin-based NP formulation of paclitaxel) conjugated with different tumor-homing peptides that selectively recognize tumor blood vessels or tumor lymphatics were tested for targeting cancer cells in mice with breast tumors. Immunofluorescence analysis was performed in tissue sections using antibodies against CD31, podoplanin (to label lymphatic vessels), p32 (the target molecule of one of the tumor-homing peptides used), and T7 phage (associated to one of the tumor-homing peptides used), which were revealed with Alexa^®^ 594-conjugated secondary antibodies. The results demonstrated that some peptide-conjugated NPs were able to extravasate into tumor tissue, thus inhibiting tumor growth [64].

The antitumor effect of superparamagnetic iron oxide NPs, loaded with transarterial chemoembolization and Gly-Arg-Gly-Asp-Ser-Pro integrin inhibitor, was evaluated in a rat hepatocellular carcinoma model by using anti-matrix metalloprotein 9 and anti-VEGF antibodies revealed by an alkaline phosphatase supervision polymer system. Immunostaining demonstrated lower expression of both proteins in treated cells, thus suggesting reduced angiogenesis and metastasis [65].

Silica-gold nanoshells were used to apply NP-mediated photothermal cancer therapy in colon tumor-bearing mice, using 18F-FDG (fluorodeoxyglucose) positron emission tomography combined with computed tomography for early evaluation of treatment effects. Immunohistochemistry performed on tumor tissue sections for CD31 and glucose transporter 1 (as a biomarker for 18F-FDG uptake) using antibodies revealed by HRP labelled-probes and DAB showed vasculature damage and decreased 18F-FDG uptake in tumor tissue, confirming the efficacy of the treatment as well as the suitability of the monitoring technique [66].

Tumor accumulation, extravasation, and circulation profiles as a function of particle matrix, shape, and size were compared in various NPs (gold-, iron-, polymer-based NPs, carbon nanotubes, quantum dots). To this aim, immunohistochemistry using biotinylated probes for collagen IV, F4/8 (expressed in most resident tissue macrophages), VEGF, lymphatic vessel endothelial hyaluronan receptor-1, and CD31 allowed to recognize tissue components and monitor NPs biodistribution in murine lung tumor slices [67].

In an attempt to develop a strategy to allow therapeutic agents to cross the blood–brain barrier, the suitability of the intranasal route to administer plasmid DNA NPs encoding human glial-derived neurotrophic factor protein (hGDNF, able to transfect both neurons and astrocytes) fused with enhanced green fluorescent protein (eGFP) to central nervous system cells was verified. Immunofluorescence was applied to detect the distribution and pattern of cellular transfection in rat brain sections by using antibodies recognizing eGFP associated with rat endothelial cell antigen-1, glial fibrillary acidic protein (GFAP), neuronal nuclear protein, and tyrosine hydroxylase, then revealed with Alexa Fluor^®^ 647- and Alexa Fluor^®^ 488-conjugated probes. Immunohistochemistry demonstrated successful transfection and transgene expression in rat brain, transfected cells being preferentially located close to capillaries [68]. Immunohistochemistry for GFAP in mouse brain slices demonstrated a decreased cellularity of glial cells, contributing to assess the efficacy of bilayered poly(D,L)-lactide-co-glycolide nanofibrous membranes in the sequential and sustained release of chemotherapeutic and antiangiogenic agents [69].

Some nanomedical studies explored innovative treatments for diseases characterized by an inflammatory status. Different matrix mixtures of nanomicelles and nanoemulsions containing aceclofenac and capsaicin were administered topically in a murine psoriatic model: immunohistochemistry using biotinylated probes for IL-23, a cytokine playing an important role in the inflammatory response, allowed to verify the efficacy of some of the tested matrices for treatment of skin inflammation [70]. The potential of protamine–oligonucleotide NPs or liposomes coated with adiponectin to target atherosclerotic lesions in the arterial wall of mice was verified by immunofluorescence with antibodies for CD68, a marker for macrophages that occur in large amounts due to the chronic inflammation of the vascular wall, revealed with Alexa Fluor^®^ 488-conjugated probes. The colocalization of the nanoconstructs with CD68 demonstrated their internalization into the macrophages occurring in atherosclerotic lesions [71].

Particular attention was paid to eye pathological conditions. Chitosan, (poly{[(cholesteryl oxocarbonylamido ethyl) methyl bis(ethylene) ammonium iodide] ethyl phosphate}), and magnetic NPs were tested for gene delivery in the eye. After their intraocular injection in rabbits, the induced expression of GFP protein was evaluated by immunohistochemistry on frozen sections at both light microscopy (using biotinylated probes) and TEM (using colloidal gold particles). In addition, GFP and *Discosoma* red fluorescent (DsRed) protein expression was evaluated at fluorescence microscopy [72]. Immunofluorescence using Cy-3-tagged probes to detect ionized calcium binding adapter 1 molecule (a microglia/macrophage cell marker) was essential to verify the capability of Cy5-conjugated dendrimers to target activated microglia and macrophages in the retina after systemic or intraocular injection in an ischemia/reperfusion mouse model [73]. A rat model of suture-induced corneal neovascularization was treated by subconjunctival injection with nanopolymeric micelles loaded with celastrol, a traditional Chinese medicine. Corneal macrophage infiltration was evaluated ex vivo by immunohistochemistry using an anti-CD68 antibody, proving the anti-inflammatory properties of these NPs [74].

In the field of regenerative nanomedicine, immunofluorescence detection of α-smooth muscle actin, as a marker of newly formed capillaries with pericytes, was used to assess the angiogenic potential of recombinant bacteriophages displaying vascular endothelial growth factors integrated into a collagen matrix subcutaneously implanted in mice [75]. GFAP was fluorescently immunolabelled with Dylight^®^ 488-conjugated probes to assess the effects of silk fibroin nanoscaffolds on glial scar formation in a murine photothrombotic model of focal stroke, demonstrating that treated animals showed a lower astrogliosis, thus promoting recovery of functions after stroke [76]. Immunofluorescence with antibodies directed against glucocorticoid receptors and revealed with Alexa^®^ 594-conjugated probes demonstrated in mouse cochlear hair cells the efficacy of polyethylene glycol-coated polylactic acid NPs in encapsulating betamethasone phosphate administered systemically to mice after acoustic trauma. In fact, the higher translocation of glucocorticoid receptors into the cell nucleus testified their activation in hair cells [77].

A nanomedical approach was explored even to treat depression. Immunofluorescence with FITC-labelled antibodies was used to investigate the antidepressant effects of solid lipid NPs encapsulating dexanabinol and curcumin in a mouse model of major depression. This demonstrated an increased expression of cannabinoid receptor 1, phospho-mitogen-activated protein kinase 1, and phospho-extracellular signal-regulated kinase 1/2 in slices from different brain regions, thus suggesting that these factors involved in neurotransmitter modulation may improve the amount and availability of neurotransmitters and counteract depression [78].

### 4.3. Both In Vitro and Ex Vivo Applications

In some studies, immunohistochemical techniques were applied to both cultured cells and tissue samples.

Various nanoconstructs were explored for their antitumor potential. CRLX101, an NP–drug conjugate containing camptothecin, was found to improve chemoradiotherapy both in colorectal cancer cell lines and in a mouse xenograft models of rectal cancer. This finding was corroborated by immunofluorescence with antibodies conjugated with Alexa Fluor^®^ 568 or Alexa Fluor^®^ 594 for γH2AX (a phosphorylated form of H2A histone family member X used as a marker of DNA double-strand DNA breaking and repair), hypoxia-inducible factor 1-α, carbonic anhydrase IX (a diagnostic marker for various cancers), and caspase-3. It was in fact demonstrated that CRLX101 significantly inhibited DNA repair and hypoxia-inducible factor 1-α pathway activation in tumor cells [79].

Gd^3+-^doped WS_2_ nanoflakes modified with polyethylene glycol were set up to improve efficient tumor homing, allowing trimodal photoacoustic/computed tomography/magnetic resonance imaging, as well as photothermal and radiation therapy. Their effects were tested in a murine breast cancer cell line and DNA damage was revealed by immunofluorescence for γH2AX using antibodies labelled with Cy633. After intravenous injection of nanoflakes in a murine model of subcutaneous breast tumor, immunofluorescence for CD31 and pimonidazole (a hypoxia marker) was performed in tumor tissue slices using antibodies labelled with rhodamine and Alexa^®^ 488, respectively [80].

As a regenerative approach, carbon nanotubes impregnated with subventricular zone neural progenitor cells were transplanted by microinjection in a rat model of focal cerebral ischemia. Before transplantation, progenitor cells proved to be able to differentiate into neurons and astrocytes thanks to immunofluorescence evidence obtained with antibodies directed against a neuronal marker (neuron-specific class III β tubulin) and an astrocyte marker (GFAP), and revealed with FITC- and rhodamine-conjugated probes, respectively. After transplantation, immunofluorescence in brain tissue slices allowed to detect the bromodeoxyuridine (BrdU)-labelled transplanted cells with an anti-BrdU antibody revealed with rhodamine. Moreover a panel of antibodies revealed with biotinylated probes visualized using Zymed^®^ Histostain-Plus Bulk kit + DAB or Fast-Red for light microscopy, and FITC or rhodamine for fluorescence microscopy was used to monitor the fate of BdrU-labelled cells, namely, nestin (a marker for neural progenitor cells), microtubule-like associated protein 2 (a marker for fully differentiated neurons), neuron-specific class III β tubulin, GFAP, Ki-67, CD11b/c (a marker for reactive microglial cells), synaptophysin (a marker of synapse formation), and doublecortin (a marker of neuronal precursor cells and immature neurons). The results showed that carbon nanotubes impregnated with neural progenitor cells can improve healing of stroke damage [81].

NPs were also explored for their suitability as vaccine vectors. The efficiency of the chitosan-encapsulated DNA-based respiratory syncytial virus vaccine was evaluated by detecting, in vitro (Cos-7 and HEp-2 cell lines) and in vivo (organ slices of immunized mice), the respiratory syncytial virus by immunofluorescence using FITC-labelled probes or by immunohistochemistry using biotinylated antibodies revealed with 3′-amino-9′-ethylcarbazole [82].

## 5. Combination of Immunohistochemistry and Histochemical Staining in Nanomedical Studies

In several nanomedical studies the histochemical approach was composite including the application of both labelled antibodies and histochemical stains.

### 5.1. In Vitro Applications

Among the studies based on in vitro models, many are designed to set up nanotools with antitumor properties.

Apoptosis and necrosis of cultured cancer HeLa cells were detected by immunohistochemistry for caspase-3 at bright microscopy using biotinylated antibodies as well as by fluorescent DNA staining (4′,6-diamidino-2-phenylindole also known as DAPI, Hoechst, propidium iodine) to evaluate the efficacy of treatment with (poly(3-hydroxybutyrate-co-3-hydroxyhexanoate)-based NPs containing the antineoplastic agent etoposide [83].

Primary cultures of mouse bone marrow mononuclear cells were treated with Resovist^®^ (a superparamagnetic iron oxide NPs) and characterized by flow cytometry using a panel of antibodies recognizing CD133 (a cancer cell marker), CD31, CD34 (a marker for human hematopoietic stem/progenitor cells), CD45 (a marker for hematopoietic cells), kinase domain receptor, and F4/80 (a marker for murine macrophages). The phagocytic activity was histochemically assessed by using 1,1-dioctadecyl-3,3,3,3-tetramethylindocarbocyanine-labelled acetylated low-density lipoprotein followed by FITC-labelled *Ulex europaeus* agglutinin. Moreover, Pussian blue staining was used to detect the NPs inside the cells at light microscopy, while antibodies against the von Willebrand factor and CD31, the kinase domain receptor, were used for immunofluorescence using FITC-labelled probes. The histochemical evidence demonstrated the suitability of Resovist^®^ to efficiently enter mouse bone marrow-derived endothelial progenitor cells and to image them with magnetic resonance imaging, opening promising perspectives for tracing these cells in vivo after transplantation for, e.g., vascular disorders or cancer treatment [84].

An albumin-gold NP bionanosystem for selective targeting of albondin receptors (located on the membrane of malignant liver cancer cells) was tested for its efficacy in enhancing laser thermal ablation in a mouse hepatocellular carcinoma cell line. The annexin V-Cy3 apoptosis detection assay was used to assess the apoptotic rate at fluorescence microscopy, while albondin receptors were localized by immunofluorescence using Cy3-labelled probes, demonstrating that NPs selectively enter cells via albondin receptors and are responsible for apoptosis initiation following photothermal treatment [85].

Nanocomplexes made of superparamagnetic iron oxide NPs and an antisense oligonucleotide targeting the transcription regulator MAX dimerization protein 3 (MXD3, highly expressed in high-risk neuroblastoma) were developed to improve neuroblastoma treatment. The nanocomplexes were tested in vitro in two human neuroblastoma cell lines, and immunofluorescence for MXD3 using Alexa^®^ 488-labelled probes showed a drastic decrease in this factor in treated cells, while the apoptotic rate, evaluated using FITC-labelled annexin V and propidium iodide, increased [86].

In the frame of nanomedical research aimed at treating HIV infection, the uptake, endocytic trafficking, and release of fluorescently-labelled-nanoformulated crystalline antiretroviral NPs were investigated in human monocyte-derived macrophages. Immunofluorescence analysis was performed using a panel of antibodies (such as clathrin, early endosome antigen 1, lysosome-associated membrane protein 1, transferrin, Ras-related proteins) directed against the endosomal compartment and revealed with Alexa Fluor^®^ 488-, 594-, and 647-labelled probes. Moreover, pHrhodo-dextran conjugate dye for phagocytosis was used to visualize pH level inside endosomes. Finally, the antiretroviral efficacy of native and released NPs was tested by immunohistochemistry in HIV-1-infected cells using antibodies recognizing HIV-1 p24 visualized with HRP-labelled probes and DAB staining. Findings demonstrated that NPs entered the cells via clathrin-dependent endocytosis, underwent endosomal escape bypassing the lysosomal degradation, and retained complete antiretroviral efficacy [87].

A combined histochemical approach was also applied to set up nanotools for reconstructive purposes. A composite scaffold made of macroporous polycaprolactone embedded with a porous matrix composed of chitosan, nanoclay, and β-tricalcium phosphate was tested for its suitability to deliver anthracycline and promote formation of mineralized matrix in human bone marrow-derived mesenchymal stem cells. The osteoinductive potential of the scaffold was demonstrated by the von Kossa staining and the histochemical staining for alkaline phosphatase, as well as by detecting osteocalcin immunohistochemically using biotinylated antibodies revealed with peroxidase-conjugated streptavidin [88].

The influence of Ti-6Al-4V alloy nanotubes on cell adhesion and differentiation was investigated in a human osteosarcoma cell line by applying histochemical methods such as a live/dead^®^ viability assay for fluorescence microscopy, as well as by immunofluorescence for talin and vinculin as markers of cell adhesion, and type I collagen, osteopontin, osteocalcin, and placental alkaline phosphatase as proteins involved in osteogenic differentiation. Alexa Fluor^®^ 488 was used as fluorophore to detect the primary antibodies. This approach allowed to determine the anodization conditions suitable to ensure optimal nanotube adhesion to cells [89].

Electrospun nanofibrous membranes made from poly(l-lactide) modified with a thin fibrin nanocoating were produced to improve wound healing and skin regeneration. When the membranes were tested in vitro on cultured human dermal fibroblasts, immunofluorescence analysis for fibrinogen using Alexa Fluor^®^ 488-labelled antibodies demonstrated the long-term stability of the fibrin coating, while antibodies against the β1-integrin chain and collagen I showed higher adhesion and collagen production in cells grown on fibrin-coated membranes. Moreover, staining with Texas Red C2-maleimide (that stains the cytoplasm), tetramethylrhodamine-conjugated phalloidin (for F-actin), and Hoechst (that stains nuclear DNA) showed the progressive spreading of fibroblasts on the nanofibrous membranes [90].

Single-walled carbon nanotube-collagen scaffolds were tested as growth supports for the efficient formation and function of intercalated discs in primary cultures of neonatal rat ventricular cardiomyocytes. Histochemical staining with calcein AM and ethidium homodimer-1 allowed for the discriminating of live and dead cells, actin filaments were stained with fluorochrome-labelled phalloidin, and intracellular calcium transients were measured with the calcium ion indicator, fluo-4 AM. Moreover, immunofluorescence was applied to detect troponin I, sarcomeric actinin, connexin-43, N-cadherin, plakophilin2, and plakoglobin using Alexa Fluor^®^ 488- and Alexa Fluor^®^ 548-conjugated antibodies. These nanosystems were able to enhance intercalated disc assembly and functionality (especially gap junctions) by upregulating the electrical and mechanical junction proteins [91].

With the aim of designing multifunctional graphene-based devices as therapeutic tools for the central nervous system, the effects of graphene oxide nanosheets on neuronal cells have been investigated in primary rat hippocampal and cortical cultures. Calcium imaging was performed by using the fluorescent dye Fura-2-AM, while depolarization-dependent staining of synaptic terminals was obtained with the styryl dye N-(3-triethylammoniumpropyl)-4-(4-(dibutylamino)styryl)pyridinium dibromide (FM1-43). Immunofluorescence labelling of β-tubulin III, GFAP, and vesicular glutamate transporter was also carried out using Alexa Fluor^®^ 488- and Alexa Fluor^®^ 548-conjugated antibodies. The results demonstrated the capability of graphene oxide nanosheets to downregulate synaptic activity without affecting cell viability [92].

### 5.2. Ex Vivo Applications

The association of immunohistochemistry and histochemical stains has been frequently applied to tissue samples from in vivo models.

Looking for novel antitumor devices, soybean phospholipid-encapsulated MoS_2_ nanosheets were tested as photothermal agents for tumor regression. Their efficacy was evaluated by immunohistochemical staining for CD31, Ki-67, and terminal deoxynucleotidyl transferase dUTP nick end labelling (TUNEL) assay in mouse breast tumor sections, demonstrating that these nanosheets were able to kill tumor cells and significantly inhibit tumor growth [93].

Curcumin-loaded pluronic nanomicelles and curcumin-loaded poly(lactic-co-glycolic acid) NPs were tested in Ehrlich ascites carcinoma-bearing mice, and their antitumor efficacy was evaluated by immunostaining of the proliferating factors Ki-67 and B-cell lymphoma 2 (Bcl2), and the apoptotic factor caspase-3 using antibodies revealed by HRP-streptavidin–biotin + DAB. The trypan blue exclusion test was used to generally assess cell death [94].

A biotic/abiotic hybrid system consisting in nanophotosensitizer (indocyanine green)-loaded polymeric NPs attached to the surface of *Salmonella typhimurium* YB1 was set up to achieve precise tumor targeting and elimination. The nanosystems were injected in a mouse subcutaneous model of bladder cancer, and tumor tissues were analyzed to explore the photothermal-assisted bioaccumulation efficiency by combining the NPs’ detection by the indocyanine green fluorescence with immunofluorescence (using antibodies directed against the YB1 receptor revealed with Alexa Fluor^®^ 594), the histochemical staining for hypoxic cells (with the Hypoxyprobe^TM^-1 assay), and the nuclear DNA staining (with DAPI). This nanosystem proved to target hypoxia in solid tumors, significantly enhance the effect of photothermal treatment, and provide efficient fluorescence imaging [95].

As anti-inflammatory nanoconstructs, silver NPs were explored in a mouse model of allergic airway disease, and lung and trachea sections were investigated for mucin production with the classical periodic acid-Schiff (PAS) reaction and the immunostaining of mucin 5AC with peroxidase-conjugated antibodies. This demonstrated that silver NPs were able to suppress mucus hypersecretion [96].

Moreover, the intestinal inflammatory reaction induced by ischemia/reperfusion injury was found to be remarkably mitigated after simvastatin-loaded poly(ethylene glycol)-b-poly(gamma-benzyl l-glutamate) NPs’ preconditioning. In fact, immunohistochemical labelling of inflammation regulators (such as the bone morphogenetic protein 4, cyclooxygenase-2, and p38 mitogen-activated protein kinase) with biotinylated antibodies showed lower values in rat intestinal tissues treated with NPs compared to the untreated controls. Moreover, 2′,7′-dichlorodihydrofluorescein diacetate staining revealed lower production of reactive oxygen species in the same samples, as a further sign of reduced inflammation [97].

The gastroprotective activity of nanostructured lipid carriers loaded with the natural therapeutic compound, thymoquinone, was evaluated in a rat model of gastric ulcer, after oral administration of the nanoconstructs as follows: histological ulcer sections were submitted to the PAS reaction and immunolabelled for the heat shock protein 70 with peroxidase-conjugated antibodies. These nanocarriers induced an expansion of the mucous layer in the gastric wall and modulated heat shock protein 70, thus exerting an anti-ulcer action [98].

Various studies have been devoted to set up nanodevices for regenerative purposes. To evaluate the promotion of wound healing by silver NP/chitosan oligosaccharide/poly(vinyl alcohol nanofibers, a rat skin wound model was established and the skin biopsies were treated with Masson’s trichome staining to distinguish connective tissue or submitted to immunofluorescence for collagen type I and type III, thus demonstrating an increase in extracellular matrix deposition [99].

A scaffold of biodegradable polyester poly-ε-caprolactone nanofibers was developed to promote abdominal fascia healing, and tested in rabbit to reinforce abdominal closure and prevent the formation of incisional hernias. Verhoeff’s hematoxylin and green trichrome were used to stain the connective tissue in fascia slices, while immunohistochemistry for α-smooth muscle actin and CD31 with peroxidase-conjugated antibodies allowed the labelling of smooth muscle cells and endothelial cells, respectively. The nanostructured scaffold proved to increase smooth muscle cells and microvessels, positively influencing the biomechanical properties of the regenerating fascia [100].

A nanomaterial composed of hydroxyapatite nanocrystallines embedded in a porous silica gel matrix was examined in sheep bearing bone defects. Sections of tibial metaphysis were submitted to Goldner–Masson’s trichrome staining (to morphologically visualize new bone formation) and an enzyme histochemical reaction for tartrate-resistant acid phosphatase (to detect osteoclasts and mononuclear precursor cells). In addition, immunohistochemistry was applied to detect osteocalcin as a non-collagenous matrix protein expressed by osteoblasts using biotinylated antibodies revealed by avidin–biotin–enzyme complex followed by DAB. Findings demonstrated an early and efficient osteogenetic activity induced by the nanosystem in large bone defects [101].

Pegylated NP coating of murine Langerhans islets was experimented as a strategy to promote islet transplantation and survival in diabetic mice. Before transplantation, islets were maintained in vitro and cell viability was monitored by SytoGreen^TM^ 13 and ethidium bromide staining (dead cells are stained red and live cells are green), while insulin expression was analyzed by immunofluorescence using an anti-insulin antibody revealed with a phycoerythrin-labelled probe. These NPs were able to improve both ex vivo survival and insulin production in isolated islets [102].

Nanomedical research also focused on liver fibrosis. Immunofluorescence was applied to liver sections of a rat model of liver fibrosis to reveal tumor growth factor-β1, Gli-1 (a zinc finger protein also known as glioma-associated oncogene), and α-smooth muscle actin as markers of disease progression by using antibodies revealed with a DyLight^®^ 488 probe. This demonstrated that all these proteins decreased after systemic administration of polymeric NPs loaded with vismodegib and rosiglitazone, two inhibitors of the major signalling pathways involved in fibrosis pathogenesis. Accordingly, Masson’s trichrome staining of liver sections from NP-treated rats showed a significant reduction in collagen accumulation (that typically occurs during fibrosis progression) [103].

### 5.3. Both In Vitro and Ex Vivo Applications

Numerous have studies combined histochemical staining and immunohistochemistry to evaluate the effects of novel nanoconstructs both in cultured cells and biopsy samples from animal models.

In the frame of antitumor research, cisplatin-conjugated gold NPs combined with magnetic resonance-guided focused ultrasound were experimented as a therapeutic approach for glioblastoma, using various cell lines and a mouse tumor model. Immunofluorescence for γH2AX (using Alexa^®^ 488-labelled antibodies) associated with DAPI counterstaining for nuclei, fluorochrome-labelled phalloidin (Alexa Fluor^®^ 594 phalloidin), and silver enhancement to improve NP visibility allowed to demonstrate a higher DNA damage in cells treated with NPs in vitro. Consistently, in tumor slices from mice after NP systemic treatment, decreased cell proliferation and increased apoptosis were demonstrated by the immunohistochemical detection of Ki-67 and caspase-3 [104]. Another nanomedical approach to treat glioblastoma was based on polyionic complex nanomicelles conjugated with the cyclic Arg-Gly-Asp peptide. Their effect on the apoptotic rate was assessed in a glioblastoma cell line by the TUNEL assay, and by immunolabelling with FITC-conjugated antibodies of caspase-3 in rat brain tumor slices, demonstrating the induction of apoptosis in both cultured cells and nervous tissue [105].

The antitumor effect of heparin–polyethyleneimine NPs delivering plasmids that express mouse survivin T34A was evaluated in colon cancer cells both in vitro and in vivo: immunostaining of CD31 with tetramethylrhodamine-conjugated antibodies allowed the visualization of blood vessels; TUNEL assay and propidium iodine were used to detect apoptosis, and β-galactosidase staining allowed the verifying of the transfection ability of these NPs. The combination of these approaches demonstrated the capability of these nanocomplexes to induce cell death and decrease angiogenesis in colon cancer tumors [106]. The antitumor potential of a biodegradable drug-delivery system set up by co-encapsulating docetaxel and LL37 (a tumor-suppressing peptide)-loaded polymeric NPs in a thermosensitive hydrogel system was tested in vitro and in vivo. After an NP-hydrogel system administration, TUNEL assay showed a drastic increase in apoptotic rate in a colon cancer cell line, while immunofluorescence for CD31 with FITC-conjugated probes showed a markedly reduced angiogenesis in tumor samples from mice bearing colorectal peritoneal carcinomatosis [107]. Polymeric NPs loaded with a pigment epithelium-derived factor gene (a powerful antiangiogenic agent) and paclitaxel were tested as an antitumor strategy for colon and lung cancers both in vitro and in a murine model of a subcutaneous tumor. The efficacy of these nanoplatforms was verified by using immunohistochemical approaches at both brightfield (using anti-CD31 antibody revealed by streptavidin–peroxidase followed by DAB) and fluorescence (using Alexa Fluor^®^ 488-conjugated antibodies against α-tubulin as a paclitaxel target) microscopy, as well as TUNEL assay demonstrating their potential in decreasing microvessel density and promoting microtubule disassembly and apoptosis in cancer cells [108].

The tumor inhibition effect of polymeric micelles loaded with folic acid and paclitaxel was tested in a human esophageal cancer cell line and in mouse subcutaneous xenograft tumors. To this aim, an annexin V/propidium iodide double-stain assay was used to assess the apoptotic rate in cultured cells, while the TUNEL assay was used on tumor sections. Moreover, the immunohistochemical detection of the apoptosis regulators, Bcl2, Bax (also known as Bcl-2-like protein 4), and caspase-3 was performed on tumor slices using biotinylated antibodies, demonstrating the capability of these micelles in inhibiting xenograft tumors [109].

Polyplexes made of polyethylene glycol-grafted polyethylenimine functionalized with superparamagnetic iron oxide NPs and a gastric cancer-associated CD44v6 single-chain variable fragment were tested as gastric cancer-targeting and magnetic resonance imaging (MRI)-visible NPs for the delivery of siRNA. The cellular uptake and distribution of these antibody-directed polyplexes were analyzed by using FITC-conjugated antibodies directed against a polyhistidine-tag in a human gastric carcinoma cell line, while their biodistribution inside mice tumors after systemic administration was revealed in Prussian blue-stained sections. These polyplexes demonstrated a good cell internalization and transfection in vitro and specific tumor targeting in vivo [110].

Immunohistochemistry (with peroxidase-labelled antibodies) and immunofluorescence (with Alexa Fluor^®^ 488-conjugated probes) for Akt (serine/threonine-protein kinase), NF-κB, and Bad (Bcl2-associated agonist of cell death) as well as TUNEL assay demonstrated in hepatoma cell lines and in mouse tumor liver sections that carnosic acid-loaded chitosan NPs were able to accelerate apoptosis thus slowing down cancer progression [111].

To improve cancer treatment and target the hypoxic tumor microenvironment, onion-ring-shaped carbon nano-onions and carbon nano-onions embedded in MnO_2_ nanosheets were developed as novel theranostic photothermal transducers. Their efficacy was tested in a papilloma cell line using peroxy orange 1, a fluorescent sensor of hypoxia, and calcein AM and propidium iodide as live and dead markers, respectively. These nanocomplexes were also injected into mice bearing subcutaneous tumor xenografts and, after photothermal therapy, tumor tissue slices were submitted to the TUNEL assay to determine the apoptosis rate, and to immunofluorescence for detecting Ki-67, hypoxia-inducible factor 1-α, N-cadherin, and epithelial cell adhesion molecule. The experimental evidence demonstrated that these nanosystems were able to downregulate the secretion of hypoxia-inducible factors interfering with cancer cell proliferation and favoring tumor angiogenesis [112].

Some nanoconstructs were investigated for their regenerative potential. Poly(lactic-co-glycolic acid) microspheres encapsulating the plasmid of human bone morphogenetic protein 2 (BMP-2)/polyethylenimine NPs were developed, to construct a delivery system for BMP-2 cDNA plasmid, to efficiently transfect target cells and induce secretion of functional BMP-2 protein to repair large bone defects. The efficacy of these nanoconstructs was tested in a mouse pre-osteoblastic cell line and in rats bearing bone defects. The Alizarin red staining was used to assess mineralization in pre-osteoblasts, while the expression of BMP-2 was assessed immunohistochemically in bone sections using peroxidase-labelled antibodies [113].

The regeneration capability of a heparin/chitosan NP-immobilized decellularized scaffold for the controlled release of VEGF was investigated in cultured endothelial cells and in subcutaneous implants in mice. The use of fluorescein diacetate as a vital stain allowed for observing endothelial cells forming tubular structures in vitro in the presence of NPs. The tissues surrounding the implants containing NPs were studied by immunohistochemistry using peroxidase-conjugated antibodies: a higher vascularization was observed thanks to the positivity for CD31, and the infiltrating cells were identified as vimentin-positive fibroblasts and CD68-positive macrophages. In addition, extracellular matrix components were analyzed using Herovici’s staining for mature collagen fibrils and Alcian blue staining for glycosaminoglycans, demonstrating an accelerated matrix remodelling in the presence of NPs [114].

Synthetic self-assembling peptide nanofiber scaffolds were used to transplant rat primary middle-ear epithelial cells: primary cultured cells transfected with eGFP transgene, and sections of the middle-ear bullae of the recipients were investigated by immunohistochemistry for cytokeratin 5, 6, 8, and 18 (as epithelial markers), vimentin (as a mesenchymal cell marker), collagen type III (as a marker of extracellular matrix component), collagen type IV (as a marker of the basement lamina), and E-cadherin (as a marker of adherens junctions) using HRP- or Cy3-conjugated antibodies In addition, sections were stained with the PAS reaction to detect mucus proteins. Results demonstrated that transplanted cells retained normal morphology and function, forming new epithelial and subepithelial layers [115].

A theranostic nanosystem consisting of magnetic iron oxide nanocubes and poly(lactide)-polycarboxybetaine accompanied with phosphatidylserine was established for early treatment of myocardial infarction. These nanosystems were tested for endosomal escape by a histochemical approach using fluorescamine-labelled phosphatidylserine and Lysotracker red in a mouse macrophage cell line, while their effects under inflammatory conditions were evaluated by immunofluorescence using eFluor^®^ 450-conjugated antibodies recognizing F4/80 (a marker for murine macrophages), CD86 (expressed in pro-inflammation macrophages), and CD206 (a mediator of endocytosis and phagocytosis, expressed by the activation of macrophages and antigen presentation). In heart tissue slices of a rat infarction model, the nanosystems were detected by Prussian blue staining while interstitial fibrosis was visualized by Masson’s trichrome stain. The combination of these techniques demonstrated accumulation of the nanosystems in infarcted area where the resolution of early inflammatory responses was accelerated [116].

The reconstructive potential in the spinal cord injury of a self-assembling peptide nanofiber scaffold was tested in transplanted neural progenitor cells and Schwann cells cultured within these scaffolds: the immunopositivity for p75 allowed for identifying Schwann cells; for nestin, the neural progenitor cells; for GFAP, the astrocytes; for Rip, the oligodendrocytes; and for β-tubulin type III, the neurons. Then, the scaffolds were transplanted into the transected dorsal column of rat spinal cord and a panel of antibodies was used to detect p75, nestin, GFAP, Rip, β-tubulin type III, NF200 (for axons), serotonin (for raphespinal axons), calcitonin gene-related peptide (for primary sensory axons), ectodysplasin A (ED1, for macrophages), and myelin basic protein (for myelin). For both in vitro and ex vivo immunostaining, Alexa^®^ 568-conjugated secondary antibodies were used. In addition, the alkaline phosphatase stain was used to monitor angioregeneration. Histochemical evidence demonstrated that this nanofiber scaffold promotes the migration of host cells and the growth of axons and blood vessels, efficiently bridging injured spinal cord [117]. The efficacy of the same nanostructured scaffold was tested in a rat model of acute brain injury by fluorescence microscopy using the TUNEL assay to detect apoptotic cells, Nissl staining for morphological analysis, and Alexa^®^ 568-conjugated antibodies to detect GFAP, ionized calcium binding adaptor molecule 1 (IBA1), and ED1 to identify astrocytes, microglia, and macrophages, respectively [118].

To combat inflammatory conditions, hydroxyl-terminated polyamidoamine dendrimer drug constructs were assembled with conjugated triphenyl–phosphonium to target mitochondria and containing N-acetyl cysteine, which was thus delivered to mitochondria. The antioxidant and anti-inflammatory effects were studied both in human macrophages and murine glial cell lines as well as in a rabbit neuroinflammation model. To visualize Cy5-labelled nanocomplex targeting in vitro, mitochondria were immunolabelled with an anti-apoptosis-inducing factor (AIF) antibody revealed with Alexa Fluor^®^ 488, while Lectin Dylight^®^ 594-stained microglia/macrophage membranes and NucBlue^TM^ revealed the cell nuclei. Cell viability was assessed by the trypan blue exclusion test. Brain sections from rabbits administered with the dendrimer complexes were submitted to immunofluorescence procedure using anti-IBA1 and anti-mitochondria antibodies labelled with Alexa Fluor^®^ 488, thus demonstrating effective targeting to the mitochondria of activated microglial cells [119].

The suitability of hydrophobic poly(methyl methacrylate) cores and branched polyethyleneimine shells as nanocarriers to deliver microRNA-loaded plasmid to macrophages was evaluated in primary cultures of Kupffer cells and in mice. The transfection of the nanocomplexes and the apoptotic rate were assayed by flow cytometry using GFP expression and annexin V-positivity, respectively; in addition, fluorescence microscopy was used to visualize GFP in cultured cells while the expression of NF-κB P65 (the target protein) was evaluated by immunohistochemistry with HPR-conjugated antibodies in liver sections of mice that were treated with the nanocomplexes by injection via the portal vein [120].

Finally, the efficiency of various antibodies-, enzymes-, or ligand-bound NPs to target specific cells for therapeutic and/or diagnostic purposes has been demonstrated in vitro and ex vivo by applying immunohistochemical techniques at bright field and fluorescence microscopy [32,121,122,123,124,125,126,127,128,129,130,131,132,133,134,135,136,137,138,139], also associating histochemical techniques at light microscopy and TEM [140,141,142].

### 5.4. In Vitro, Ex Vivo, and In Vivo Applications

Thanks to the development of in vivo imaging technology, the histochemical approach has also become suitable for living organisms in addition to the in vitro/ex vivo models.

In order to verify the efficacy of image-guided X-irradiation to increase the accumulation of iron oxide NPs in tumors, tissue slices from mice bearing subcutaneous melanoma or breast cancer were processed with a panel of antibodies for endothelium, pericytes, interstitial extracellular matrix, and cell apoptosis, then detected by biotinylated-secondary antibodies visualized with avidin/biotin complex and DAB. Moreover, GFP-labelled human breast adenocarcinoma cells, AngioSense^®^ (a near-infrared fluorescent-labelled polyethylene glycol polymer as a blood-pool agent) and fluorescent tomato lectin (a vital endothelial stain) were used to monitor NPs biodistribution and effect in vivo by optical imaging [143].

In a more recent study [144], the efficacy of a redox-responsive lipid–polymer hybrid NP platform to deliver p53-encoding synthetic mRNA has been tested in cultured Hep3B and H1299 cancer cell lines as well as in different orthotopic and disseminated mouse tumor models (the p53 tumor suppressor gene undergo alteration in various cancers). Immunofluorescence microscopy, applied in cultured cells and murine tissues to detect p53 protein as well as apoptotic (Bax and caspase-3) and proliferation markers (Ki-67 and proliferating cell nuclear antigen) with Alexa Fluor^®^ 647-conjugated probes, was associated with various histochemical staining, such as DAPI for nuclear DNA, Lyso Traker^TM^ for endosomes, or annexin V-propidium iodide for apoptotic cells. In addition, mRNA-containing NPs were labelled with Cy5 or eGFP to allow their detection inside cells and tissues at confocal microscopy, while luciferase-expressing Hep3B cells were used to monitor tumor growth in the liver of murine models by bioluminescent in vivo imaging. The combination of these histochemical and immunohistochemical techniques allowed to demonstrate that this NP platform was able to restore the tumor-suppressing p53 gene with significant antitumor effects.

Nanoconstructs were also investigated for their capability to deliver anaesthetics. The low toxicity and the efficacy of mesoporous organosilica NP-based platforms designed for the acidity/ultrasound controlled and sustained release of loaded ropivacaine for local anaesthesia was investigated in a mouse model [145]. The fluorophore Cy5 was coupled to the nanoplatform to visualize the release of ropivacaine in vivo by optical imaging, while immunofluorescence was applied using FITC- and Cy3-labelled probes to dorsal root ganglia to detect the transient receptor potential cation channel vanilloid subtype 1, which is a non-selective cation channel involved in tissue injury-induced hyperalgesia and painful stimuli integration, and c-Fos as a marker protein for the activation state of the cell, thus revealing the effect of the analgesic on ganglion neurons.

## 6. Conclusions

From the above overview of scientific articles, it is evident that histochemistry has been playing a leading role in nanomedical research. Whatever the purpose for which the nanoconstructs had been designed or the experimental model(s) chosen to test them, histochemical techniques have largely been applied to single cells, tissue slices, and even living animals.

Histochemical stains and labelled antibodies were used separately or in association, although immunolabelling appears as the mostly used histochemical approach, due to its extremely specific molecular targeting. In fact, antibodies against specific NP components allow unequivocal nanoparticulate localization in the biosystem; antibodies against tumor markers are essential to reveal their distribution and expression following NP treatment; antibodies against markers of cell functional states (e.g., proliferation, activation or apoptosis) allow for evaluating the impact of NP on cell activity; cell-type specific antibodies make it possible to elucidate the involvement of different cell lineages in the NP-related histological modifications. On the other hand, long-established histochemical methods such as Alcian blue, the PAS reaction, or Masson’s trichrome staining were often used to identify histological components and monitor their modifications after NP treatment.

The light microscope (both in bright field and fluorescence) was (and still is) the instrument of choice to observe the results of histochemical approaches, whereas TEM was more rarely used (being almost exclusively devoted to the ultrastructural detection of unstained NPs). This is not surprising because light microscopy requires relatively simple and rapid experimental procedures, and offers the possibility to examine wide cell populations or large tissue samples; moreover, although the resolution is about 200 nm, it generally provides enough information to understand NP distribution and effects. Conversely, TEM requires complex and time-consuming sample processing, and observations can be made on a very small-sized sample; in addition, its high resolution (about 2 nm in conventional TEM) often goes beyond the real needs for visualizing a histochemical staining. However, it is worth noting that histochemical techniques applied at light microscopy are unable to allow the visualization of single NPs but only NP clusters can be detected because of the lower resolution of this tool. For the same reason, at light microscopy, it is impossible to distinguish between native and degraded NPs. At present, the only tool able to identify NPs individually is TEM, and this limitation should be taken into account when evaluating NP size, density, or structural integrity.

In conclusion, in browsing the scientific literature, it is apparent that histochemical techniques are, often unconsciously, used by scientists who work in cutting-edge research and look at histochemistry as an auxiliary discipline that is not even worth being mentioned. As Raymond Coleman wrote [146], “histochemistry is often mistakenly perceived as an archaic discipline, and its contributions to cell and molecular biology are not always given the credit it deserves”. Quite the opposite, histochemistry provides an irreplaceable toolkit for researchers in the biomedical field, as clearly demonstrated by nanomedical research. It may be foreseen that histochemical techniques will increasingly be used to understand the functional relationships between nanoconstructs and the biological environment: traditional stains will be rediscovered for novel applications, new probes will be developed, and techniques so far seldom used (such as enzyme histochemistry and in situ hybridization) will become more popular. Moreover, histochemistry is gaining attention in imaging techniques able to provide novel information such as super-resolution microscopy (which brought the resolution of fluorescence microscopy to the nanoscale), or optical imaging (which has opened the way to the dynamic, real-time detection of specific molecules in living organisms), or mass cytometry (by which dozens of antigens may simultaneously be detected in a single sample using rare-earth-metal isotopes). No doubt, histochemistry will continue to be helpful and inspiring in nanomedical research.

## Data Availability

Data sharing is not applicable.
